# Role of endothelial nitric oxide synthase and endothelin-1 polymorphism genes with the pathogenesis of intraventricular hemorrhage in preterm infants

**DOI:** 10.1038/srep42541

**Published:** 2017-02-13

**Authors:** Dawid Szpecht, Janusz Gadzinowski, Agnieszka Seremak-Mrozikiewicz, Grażyna Kurzawińska, Marta Szymankiewicz

**Affiliations:** 1 Chair and Department of Neonatology, Poznan University of Medical Sciences, Poland; 2 Department of Perinatology and Women’s Diseases, Poznan University of Medical Sciences, Poznan, Poland

## Abstract

In the pathogenesis of neonatal intraventricular hemorrhage (IVH) in preterm infants, an important role is played by changes in venous and arterial cerebral flows. It has been shown that the ability of autoregulation of cerebral flows in response to variations in arterial blood pressure in preterm infants is impaired. This impaired autoregulation causes an increased risk of germinal matrix rupture and IVH occurrence. We examined three polymorphisms of genes, related to regulation of blood flow, for an association with IVH in 100 preterm infants born from singleton pregnancy, before 32 + 0 weeks of gestation, exposed to antenatal steroids therapy, and without congenital abnormalities. These polymorphisms include: eNOS (*894G* > *T* and *−786T* > *C*) and EDN1 (*5665G* > *T* ) gene. We found that infants with genotype GT eNOS *894G* > *T* have 3.4-fold higher risk developing of IVH born before 28 + 6 weeks of gestation. Our investigation did not confirm any significant prevalence for IVH development according to eNOS *−786T* > *C* genes polymorphism. Our novel investigations in EDN1 *5665G* > *T* polymorphism did not show any link between alleles or genotypes and IVH. Future investigations of polymorphisms in blood-flow associated genes may provide valuable insight into the pathogenetic mechanisms underlying the development of IVH.

Intraventricular hemorrhage (IVH) is the most common form of bleeding into the central nervous system in neonates; its occurrence is significantly increased in a group of very low birth weight (VLBW) preterm neonates born before 32 weeks of pregnancy. Frequency of the IVH in VLBW has declined from 40–50% in the early 1980s to 20% in the late 1980s. In the last two decades, IVH frequency has remained stationary. In infants weighing 500–750 g, IVH occurs in about 45%^1^. In the pathogenesis of IVH in preterms, impaired ability to autoregulate fluctuating cerebral blood flow (CBF) in combination with immature, fragile blood vessels of the germinal matrix (GM) tissue, leads to hemorrhage into the ventricular system^2^. Cerebral blood flow through an infant’s brain is controlled by four mechanisms: autoregulation, chemical, metabolic and neurogenic regulation^3^. In healthy full term infants, the regulation of cerebral blood flow responds to stimuli in the same way as it does in an adult. Cerebral blood vessels, with their rich innervation (both sympathetic and parasympathetic), respond by contracting to neurotransmitters such as noradrenaline, serotonin and neuropeptide Y, and by relaxation to acetylcholine, vasoactive intestinal peptide and nitric oxide. In the pathogenesis of neonatal intraventricular hemorrhage (IVH) in preterm infants, an important role is played by changes in venous and arterial cerebral flow. It has been shown that the ability of autoregulation of cerebral flow in response to variations in arterial blood pressure in preterm infants is impaired. This impaired autoregulation increases the risk of germinal matrix rupture and IVH occurrence^4^.

The endothelin-1/nitric oxide system is involved in regulating the flow of blood through vessels. Nitric oxide (NO) works directly in vasculature to activate soluble guanylate cyclase and make cyclic GMP (cGMP). The rise in intravascular cGMP decreases intracellular Ca^2+^, and also activates protein kinase G (PKG). In the classic signaling pathway endothelin-1/nitric oxide, endothelin-1 (EDN1) stimulates endothelial nitric oxide synthase (eNOS) to produce NO. This takes place by activating the ETB receptor pathway and PI3-K/Akt, which then stimulates the phosphorylation of eNOS, to produce further NO. EDN1 binds the receptor ETA and activates phospholipase C (PLC), which catalizes the phophatidylionsitol-4,5,-bisphosphate (PIP2) to form inositol-1,4,5- triphosphate (IP3) and 1,2-diacylglycerol (DAG). IP3 diffuses to specific receptors on the surface of the endoplasmic reticulum and releases stored Ca^2+^ into the cytoplasm. By contrast DAG in the presence of calcium ions activates a protein kinase C (PKC)^2^.

As already mentioned in the classic signaling pathway of endothelin-1/nitric oxide, EDN1 stimulates eNOS to produce NO. On the other hand, NO may inhibit the synthesis and hemodynamic effects of EDN1. NO and EDN1 physiologically interact to regulate vascular tone inseparably. It seems that eNOS gene polymorphisms (*894G* > *T* or *−786T* > *C*) may increase the risk of IVH in preterm infants by significantly disrupting the regulation of cerebral flow^4^,^5^. While their impact on the development hemodynamic disturbances in newborns and IVH is unknown, research to date has shown that the polymorphic variant of the EDN1 gene: Lys198Asn plays a significant role in the pregnancy and also in the pathogenesis of arterial hypertension in adults.

The aim of this study was to evaluate the possible relationship between polymorphisms in genes encoding eNOS and EDN1 and the occurrence of IVH in a population of infants born from 24 + 0 to 32 + 0 weeks of gestation.

## Results

The median gestational age of enrolled children was 29 ± 2 weeks (range 24 + 0–32 + 0), and the median birth weight was 1170 ± 361 grams. In our study population, 45 infants developed IVH. The infants were diagnosed according to grades of IVH; 15 (33.33%) newborns were diagnosed with IVH grade I, 20 (42.22%) with grade II, 8 (17.77%) with grade III and 3 (6.66%) with grade IV IVH.

The incidence of IVH was comparable in female (20; 44.44%) and male (25; 55.56%) neonates with no significance. The incidence of IVH grade II to IV was: the higher the lower the gestational age and was significantly higher in children born from 24 + 0 to 28 + 6 weeks of gestation than born from 29 + 0 to 32 + 0 weeks of gestation (74.19% vs 25.81%); p = 0.007); the higher the lower Apgar score in first (6(1–10) vs 8(2–10); p = 0.007) and fifth minute of life (4(1–10) vs 7(1–8); p = 0.001); more often in children diagnosed with intrauterine infection (70.97% vs 47.83%; p = 0.031). In children treated due to hypotension the incidence of IVH grade II to IV is higher (51.61% vs 13.04%; p = 0.002). Similarly, it occurred more often in infants treated for acidosis (54.84% vs 27.54%; p = 0.009). All children needed ventilation support (52 children had non-invasive ventilation and 48 conventional ventilation followed by non-invasive support). Infants with IVH grade II to IV were more often conventionally ventilated (80.65% vs 33.33%; p<0.0001). Ten of 100 (10%) patients died, including 7 (70%) patients with grade II to IV of IVH. All children that died were born from 24 + 0 to 28 + 6 weeks of gestation (18.18%). Characteristic of enrolled infants is shown in Table 1.

Analysis showed higher prevalence of grade II and IV IVH in children born from 24 + 0 to 28 + 6 weeks of gestation with the genotype GT (OR 3.431; 1.049–11.22) eNOS *894G* > *T* gene polymorphism. This finding was not present in all children enrolled into our study (born from 24 + 0 to 32 + 0 weeks of gestation). Our investigation did not confirm any significant prevalence for IVH development in any other genotypes/alleles of eNOS *−786T* > *C* and EDN1 *5665G* > *T*. Genotype distribution of the polymorphisms in infants with/without IVH grade I and with IVH grade II-IV is presented in Table 2; in infants born between 24 + 0 – and 28 + 6 weeks of gestation with/without IVH grade I and with IVH grade II-IV are presented in Table 3.

The analysis did not show any higher prevalence of studied genotypes in patients with IVH grade II-IV treated for hypotension (Table 4) and acidosis (Table 5).

## Discussion

IVH is a major complication of prematurity with a multifactorial etiology, including intrinsic fragility of the GM vasculature and the disturbance in CBF^1^,^2^. The overall mechanism of GM vascular fragility included: the accelerated endothelial proliferation (angiogenesis) associated with high levels of vascular endothelial growth factor and angiopoeitin-2 and reduced expression of transforming growth factor β compared to other brain regions^6^, which are stimulated by hypoxia (GM is more sensitive to hypoxia than other brain regions)^7^; high metabolic activity (GM consisted of neuronal and glial precursors cells in various proliferation, migration and maturation)^1^,^2^; scarcity of pericytes^8^, low fibronectin levels in the basement membrane^9^ and reduced glial fibrillary acidic protein (GFAP) expression in the astrocyte endfeet^10^. Disturbances of CBF, includes fluctuating CBF and impaired CBF autoregulation ability to respond to variations in arterial blood pressure in preterm infants (pressure passivity of CBF). Asynchronized ventilation support (nowadays not used in neonatal intensive care units), hypercarbia and hypoxia, asphyxia, hypotension and its treatment, hypertension, acidosis and its treatment with rapid infusion of sodium bicarbonate may contribute to the fluctuation of CBF and correlate to IVH development^11^. A prolonged vaginal delivery, low Apgar score, recurrent tracheal suctioning and intrauterine infection may also disturb the cerebral blood flow and contribute to IVH^1^,^2^. The pressure passivity of CGF correlates with lower gestational age and birth weight^12^. In our study we confirmed, that IVH occurs more often in children: born before 28 + 6 weeks of gestation, with lower gestational age, lower Apgar score, with symptoms of infection, conventionally ventilated and treated due to hypotension and acidosis.

A number of vasoactive agents, including nitric oxide have been linked with CBF^13^. Nitric oxide is continuously synthesized in the human body, inter alia in vascular endothelium. NO is synthesized from the guanidine group of L-arginine with a release of L-citrulline in a reaction catalyzed by eNOS in the presence of molecular oxygen and required cofactors: reduced nicotinamide adenine dinucleotide phosphate (NADPH), flavin adenine dinucleotide (FAD) and tetrahydrobiopterin (BH4). The eNOS enzyme, takes up so-called cellular membrane microdomains in cells such as endothelium, myocytes, platelets and neurons, playing an important role in the transduction of signals reaching the cell from the outside, for example by interaction with caveolin-1. NO synthesis disorders may result from genetically conditioned disruptions in eNOS activity. The eNOS gene (eNOS coding gene) comprises 26 exons and 25 introns; it codes 1203 aminoacids with molecular mass of 133 kDa. The eNOS gene polymorphism whereby guanine (G) is replaced with thymine (T) at nucleotide 894 (exon 7), results in a change of the aminoacid sequence Glu298Asp. The *−786T* > *C* polymorphism of the eNOS gene replaces thymine with cytosine in the eNOS gene promoter at position 786. It is thought that in the presence of *894G* > *T* and *−786T* > *C* polymorphic variants, eNOS enzymatic activity may be impaired. In endothelium, NO plays a central role in the regulation of local blood pressure by acting as a vasodilatory agent that ensures adequate blood flow through the tissues. Moreover, it counteracts factors with strong vasoconstriction, such as endothelin 1, angiotensin II, and it inhibits aggregation and adhesion of platelets by reducing the production of platelet activation factor (PAF) by the endothelium. It also protects vessel walls by inhibiting oxidation of lipids and inactivating oxygen free radicals^14^.

The role of the eNOS polymorphisms in pathogenesis of the IVH was studied by Vannemreddy *et al*.^5^ and Poggi *et al*.^15^ Vannemreddy evaluated the association of the eNOS *−786T* > *C* polymorphism in 124 premature African American infants. They found that carrying the C allele increases twofold the risk of developing IVH. The mutant allele C was present in 15.3% of premature infants compared with 7.25% controls^5^. Poggi *et al*. did not find any association with IVH occurrence in population of 342 preterm newborns born before 28 weeks of gestation and genotypes of polymorphism eNOS *−786T* > *C* and *894G* > *T*^15^. We found that infants with genotype GT eNOS *894G* > *T* have 3.4-fold higher risk developing of IVH born before 28 + 6 weeks of gestation. It has been demonstrated that the T allele carrier (heterozygotes GT and homozygotes TT) have reduced enzyme function and decreased NO production, which might have negative effect on vessels and regulation blood flow^16^,^17^. Moreover NO play a crucial role in fetal and neonatal vessels growth and protection. We postulate that unfavorable genotypes may disturb adequate vessel development and blood flow. Poggi *et al*. did not find any genotype of polymorphism eNOS *894G* > *T* that may increase risk of IVH development. However, children included in their study group, were those whose mother’s did not get any antenatal steroids therapy (AST) before delivery (29% enrolled infants) and twins. AST is given in pregnant women at risk of premature delivery; aiming to accelerate fetal lung maturation and therefore reducing the incidence and severity of respiratory distress syndrome (RDS). Moreover, many studies agreeably show that it reduces overall neonatal mortality (RR 0.69; 95% CI 0.58–0.81), it enhances circulatory stability in preterm neonates, resulting in lower rates of cerebroventricular haemorrhage (RR 0.54; 95% CI 0.43–0.69), necrotizing enterocolitis (RR 0.46; 95% CI 0.29–0.74), and systemic infections in the first 48 hours of life (RR 0.56; 95% CI 0.38–0.85)^18^. The retrospective analysis of 267 preterm infants born from 24 to 32 weeks of gestation published by us confirmed that a lack of AST caused a twofold increase risk of IVH^19^. Additionally, corticosteroid stimulation of developmentally regulated gene expression and physiologic functions result lung maturation and maturation of some other tissues, including vessels^20^. *In vitro* studies have shown that steroids upregulate GFAP. Paucity of GFAP in perivascular endfeet of the germinal matrix vasculature may contribute to its fragility^21^. Based on that information we decided to exclude children born from mothers without AST to minimize risk factors for IVH in our study group. Similarly, we exclude children born from twin pregnancies due to increased risk for IVH in this group of patients^22^. We evaluated the possible association between eNOS gene polymorphism and IVH in a homogenous Caucasian population of preterm infants born before 32 weeks of gestation with minimized risk factors of IVH development. At this point, we should mention that allele C of polymorphism 786T > C is seen most common in Caucasians compared to Africans or the African population. Similarly, the highest frequency of allele T of polymorphism 894G > T is in Caucasians, and very low in African Americans and almost absent among Africans^23^.

EDN1gene polymorphism has not been studied in preterm infants yet. EDN1is the strongest known vasoconstrictor – the vasospasm effect lasting for 45–60 minutes. EDN1may play a role in maintaining hemodynamic homeostasis by changing the distribution of blood in the system^24^. It is also indicated in the literature that childbirth is a stress factor leading to an increased synthesis of EDN1 by umbilical vein endothelium. EDN1 may play a significant role in the regulation of blood flow through the feto-placental unit and brain. It is suggested that the fetus itself is also synthesizing the hormone, as a result of hemodynamic and metabolic changes occurring during uterine contractions in childbirth. It is likely that an increased concentration of EDN1in umbilical blood plasma during childbirth leads to the contraction of umbilical vessels after delivery, which may be one of the mechanisms that prepare the fetus for taking the first breath^25^. Plasma endothelin-1 concentrations were similar in preterm and term infants studied by Kuo Cy^26^ and Stefanov G *et al*.^27^ Newborns with intrauterine growth restriction, however, correlated negatively with gestational age^27^. El Sayed *et al*.^28^ and Benjamin *et al*.^29^ showed that endothelin -1 level was increased in newborns with respiratory distress syndrome. Mei Mei *et al*. found that the T allele at rs2070699 polymorphism of EDN1gene was significantly associated with an increased risk of pulmonary hypertension, higher plasma levels of EDN1and a longer ventilation time under respiratory distress^30^. EDN1concentration was significantly increased among newborns with sepsis caused by hemoculture-positive bacteria^31^. Role of *5665G* > *T* polymorphism EDN1 is unknown and have not been investigated before. Polymorphism *5665G* > *T* EDN1consists of replacing guanine for thymine on position 5665, which affects the amino acid sequence (Lys198Asn). Abnormal production of EDN1in individuals with genotype GT and TT *5665G* > *T* polymorphism may lead to impaired regulation of vascular smooth muscle tension and movement, including cerebral vessels. There were no statistical differences in allele frequency of EDN1 *5665G* > *T* according to ethnicity^32^. We could not, confirm any role of the EDN1 *5665G* > *T* polymorphism in pathogenesis of IVH. None of genotypes (GG/GT/TT) and alleles increased or decreased the risk for IVH development in preterm infants.

Based on the role of NO and EDN1 in the regulation of vascular wall tension we speculate that some genotypes/alleles of eNOS *894G* > *T*, eNOS *−786T* > *C* and EDN1 *5665G* > *T* may or may not be protective for IVH development, specifically in patients treated with 0.9% NaCl and/or catecholamines due to hypotension. Hypotension and its treatment are known risk factors for IVH^19^,^33^,^34^. However we have not found any genotype of studied polymorphisms that may increase the risk for IVH development.

The infusion of NaHCO_3_ in treatment of acidosis may change the volume of blood and have an unfavorable effect on the hemodynamics of cerebral circulation and consequently increase the risk of IVH^35^. As shown in our analysis, acidosis and its treatment increased risk of IVH grade II to IV. Comparable conclusions were formulated by Rong *et al*.^33^; Randolph *et al*.^36^; Synnes *et al*.^37^. However, in newborns treated with NaHCO_3_ we could not find any genotype/alleles that increased risk for IVH occurrence.

## Conclusions

In conclusion, this study provides that infants with genotype GT eNOS *894G* > *T* have 3.4-fold higher risk of developing of IVH if they are born before 28 + 6 weeks of gestation. The first investigation of an association between the EDN1 *5665G* > *T* gene polymorphism did not confirm role of it in IVH development. The sample size is small; thus, the result requires confirmation in a larger sample. Together these results indicate that IVH is a multifactorial disease in which environmental and genetic risk factors might operate and interact through related pathways. Future investigations of polymorphisms in blood-flow associated genes may provide valuable insight into the pathogenetic mechanisms underlying the development of IVH for a new theoretical basis for disease control and prevention.

## Material and Methods

### Study population

Between 1st June 2014 and 15^th^ August 2016, 428 infants (from 24 + 0 to 32 + 0 weeks of gestation) were born at the Clinical Hospital of Gynecology and Obstetrics at Poznan University of Medical Sciences and admitted to the Department of Neonatology at Poznan University of Medical Sciences. In order to guarantee a homogenous ethnic background, all subjects were of Caucasian origin. Due to exclusion criteria, 100 infants (23.4%) took part in the study. Exclusion criteria consisted of: infants born before 24 + 0 and after 32 + 0 weeks of pregnancy, outborn infants, newborns without antenatal steroids therapy (AST), newborns with chromosomal abnormalities, inherited errors of metabolism, TORCH infections (toxoplasmosis, other, rubella, cytomegalovirus, herpes), multiple pregnancy infants or pregnancies complicated by death of one of the fetuses.

### Clinical features

The following factors that may associate with the development of IVH were studied: gender, gestational age (GA; weeks), birth weight (BW, grams); small for gestational age (SGA, defined as birth weight under 3th percentile); type of delivery (vaginal birth vs. cesarean section); birth asphyxia (defined as APGAR score less than 6 at 10 minutes and ph < 7.0 or blood base excess (BE) <−15 mmol/l in cord blood); intrauterine infection (defined as positive culture in sterile originally accompanied by clinical symptoms or pneumonia developed in 48 hours after the birth), type of ventilation support (non-invasive vs conventional), therapy in first 7 days of life with crystalloids (bolus 10–15 ml/kg) and/or catecholamines of hypotension (defined as mean blood pressure below value corresponding to neonate’s gestational age), treatment of the acidosis with NaHCO_3_ (when blood pH was below 7.2 and/or BE less than −10 mmol/l).

### IVH diagnosis

IVH was diagnosed with the use of a cranial ultrasound (10 MHZ transducer, Prosoundα7 Premier, Aloka). Routine cranial ultrasound examinations were performed on 1^st^, 3^rd^, and 7^th^ day after birth, in accordance to local standards and recommendations in infants born earlier than 32 weeks gestation. The maximal degree of IVH was confirmed by cranial ultrasound on the 7^th^ day of life. The classification of intraventricular bleeding was based on the Papille IVH classification^38^.

### Studied polymorphisms

The criteria for selection of candidate genes in the present study were their potential involvement in the pathogenesis of vessels tension regulation and subsequent blood flow impairment in the brain.

We studied three single nucleotide polymorphisms: eNOS (*894G* > *T* and *−786T* > *C*) and EDN1(*5665G* > *T*) gene - Table 6. Samples of blood were taken right after the delivery and banked.

Genomic DNA was extracted from blood leukocytes using QIAamp DNA Blood Mini Kit (QIAGEN inc; Germany). Genotyping was performed using polymerase chain reaction (PCR) procedures. Products were analyzed by electrophoresis on 2% agarose gel with Midori Green Advanced DNA Stain (Nippon Genetics, Europe GmbH).

For detection of *894G* > *T (rs1799983*) mutation PCR was amplified with starters: F5′ AAggCAggAgACAgTggATgg A 3′ R5′ CCC AgT CAA TCC CTT TggTgC TCA 3 (PCR product 248 bp long) and hydrolyzed with MboI restriction enzyme (Thermo Scientific). The following genotypes were obtained: GG 248 bp; GT 248, 158 and 90 bp; TT 158, 90 bp.

For detection of the *−786 T* > *C* (*rs2070744*) mutation, PCR wasamplifiedwith starters: F5′ CCA CCC TgT CAT TCA gTg AC 3′ R5 TCT CTgAgg TCT CgA AAT CA3 (PCR product 296 bp long) and hydrolyzed with PdiI restriction enzyme (Thermo Scientific). The followinggenotypeswereobtained: TT 296 bp; TC 296, 220, 76 bp and CC 220, 76 bp.

The *5665G* > *T (rs5370*) polymorphismwasdetectedusing starters: F5′TCA TgA TCC CAA gCTgAAAgg CTA3′ R5′ACC TTT CTT ggAATg TTT TgA AC3′. PCR product (228 bp long) was hydrolyzed with NheI restriction enzyme (Thermo Scientific) and found the following genotypes: GG 203, 25 bp; GT 228, 203, 25 bp; TT 228 bp.

Informed consent was obtained from the parents of all infants enrolled in the study. The study followed the tenets of the Declaration of Helsinki and was approved by the Bioethics Committee of Poznan University of Medical Sciences (nr. 66/14).

### Statistical analysis

The results are presented as a percentage for categorical variables, or median (range) for non-normally distributed continuous variables as tested by the Shapiro–Wilk test. A p-value of less than 0.05 was considered significant. The Fisher exact probability test, the chi-square test, Fisher Freeman Halton and Chi-squared test with Yates correction were all used to evaluate the association between IVH and categorical variables including: gender, GA, BW, SGA, type of delivery; birth asphyxia, intrauterine infection, type of ventilation support, hypotension and acidosis therapy. Differences in non-normally distributed continuous variables were compared by the U Mann–Whitney test. Logistic regression analysis was used to compute ORs and their 95% confidence intervals (CI) for patients without IVH and IVH grade I to IV combined with different genotypes and alleles. The odds ratio (OR) and 95% confidence intervals (95% CI) were calculated in hypotensive and non-hypotensive infants with IVH grade II-IV and in infants treated due to acidosis with IVH grade II-IV in different genotype distribution of the studied polymorphisms. Statistical analysis was performed using CytelStudio version 10.0, created January 16, 2013 (CytelStudio Software Corporation, Cambridge, Massachusetts, United States), and Statistica version 10, 2011 (Stat Soft, Inc., Tulsa, Oklahoma, United States).

### Ethics statement

All procedures performed in studies involving human participants were in accordance with the ethical standards of the institutional and/or national research committee and with the 1964 Helsinki declaration and its later amendments or comparable ethical standards. The study was approved by the Bioethics Committee of Poznan University of Medical Sciences (nr. 66/14 and 799/16).

## Additional Information

**How to cite this article:** Szpecht, D. *et al*. Role of endothelial nitric oxide synthase and endothelin-1 polymorphism genes with the pathogenesis of intraventricular hemorrhage in preterm infants. *Sci. Rep.* **7**, 42541; doi: 10.1038/srep42541 (2017).

**Publisher's note:** Springer Nature remains neutral with regard to jurisdictional claims in published maps and institutional affiliations.

## Figures and Tables

**Table 1:** Baseline characteristic of enrolled infants.

	Group without IVH and IVH grade I N = 69 (%)	Group with IVH grade II- IV N = 31 (%)	P value
**Gender**			0.585^a^
Male	36 (52.17)	18 (58.06)	
Female	33 (47.83)	13 (41.94)	
**Gestational age (week)**			0.007^a^
24 + 0–28 + 6	31 (44.93)	23 (74.19)	
29 + 0–32 + 0	38 (55.07)	8 (25.81)	
**Birth weight (gram)**			0.004^a^
<750	6 (8.70)	8 (25.81)	
750–1000	15 (21.74)	12 (38.71)	
>1000	48 (69.57)	11 (35.48)	
**Cigarettes in mother**			0.987^c^
YES	6 (8.70)	2 (6.45)	
NO	63 (91.30)	29 (93.55)	
**Apgar score (Median and range)**			
1st minute	6 (1–10)	8 (2–10)	0.007^d^
5th minute	4 (1–10)	7 (1–8)	0.001^d^
**Mode of delivery**			0.148^a^
Vaginal	25 (36.23)	16 (51.61)	
Cesarean section	44 (63.76)	15 (48.38)	
**Asphyxia (ph lower than 7.0 or BE lower than −12)**			0.468^c^
YES	1 (1.44)	2 (6.45)	
NO	68 (98.56)	29 (93.55)	
**Intrauterine infection**			0.031^a^
YES	33 (47.83)	22 (70.97)	
NO	36 (52.17)	9 (29.03)	
**Ventilation support**			<0.0001
Non-invasive	46 (66.67)	6 (19.35)	
Conventional	23 (33.33)	25 (80.65)	
**Hypotension therapy**			0.0001^c^
YES	9 (13.04)	16 (51.61)	
NO	60 (86.96)	15 (48.39)	
**Acidosis therapy**			0.009^a^
YES	19 (27.54)	17 (54.84)	
NO	50 (72.46)	14 (45.16)	
**Deaths**	3 (4.41)	7 (22.58)	0.015^c^

Results are expressed as absolute number of patients (percentage) and median (interquartile range). The following tests were used: a – Chi-square test, b – Fisher Freeman Halton test, c – Chi-square test with Yate’s correction, d – Mann Whitney test. IVH – intraventricular hemorrhage.

**Table 2:** Genotype distribution of eNOS and EDN1 gene polymorphisms in infants of gestation without and with IVH or without/with IVH grade I and with IVH grade II-IV.

Genesymbol		Group without IVH and IVH I N = 69 (%)	Group with IVH grade II-IV N = 31 (%)	P value	OR (95% CI)
eNOS *894G* > *T* (rs1799983)	**Genotype**				
	GG	41 (59,42)	13 (41,94)	—	References
	GT	21 (30,43)	15 (48,39)	0,126	2,253 (0,822–6,179)
	TT	7 (10,14)	3 (9,68)	0,961	1,352 (0,196–7,038)
	**Allele**				
	G	103 (74,64)	41 (66,13)	—	References
	T	35 (25,36)	21 (33,87)	0,285	1,507 (0,740–3,022)
eNOS *−786T* > *C* (rs2070744)	**Genotype**				
	TT	31 (44,92)	7 (22,58)	—	References
	TC	32 (46,38)	19 (61,29)	0,087	2,629 (0,890–8,402)
	CC	6 (8,70)	5 (16,13)	0,158	3,690 (0,664–19,35)
	**Allele**				
	T	94 (68,12)	33 (53,23)	—	References
	C	44 (31,88)	29 (46,77)	0,0436	1,877 (0,968–3,624)
EDN1 *5665G* > *T* (rs5370)	**Genotype**				
	GG	45 (65,22)	21 (67,74)	—	References
	GT	22 (31,88)	9 (29,03)	0,976	0,877 (0,302–2,417)
	TT	2 (2,90)	1 (3,23)	1,000	1,071 (0,017–21,65)
	**Allele**				
	G	112 (81,16)	51 (82,26)	—	References
	T	26 (18,84)	11 (17,74)	1,000	0,929 (0,384–2,130)

Results are expressed as absolute number of patients (percentage). The odds ratio (OR) and 95% confidence intervals (95% CI). GG denotes homozygosity for the G-encoded eNOS *894G* > *T* polymorphism variant; TT homozygosity for the T-encoded eNOS *894G* > *T* polymorphism variant; G*T* heterozygosity for eNOS *894G* > *T* polymorphism. TT denotes homozygosity for the T-encoded eNOS *−786T* > *C* polymorphism variant; CC homozygosity for the *C*-encoded eNOS *−786T* > *C* polymorphism variant; T*C* heterozygosity for eNOS *−786T* > *C* polymorphism. GG denotes homozygosity for the G-encoded EDN1 *5665G* > *T* polymorphism variant; TT homozygosity for the T-encoded EDN1 *5665G* > *T* polymorphism variant; GT heterozygosity for EDN1 *5665G* > *T* polymorphism. EDN1 -endothelin-1; eNOS- endothelial nitric oxide synthase.

**Table 3:** Genotype distribution of eNOS and EDN1 gene polymorphisms in infants born 24–28 weeks without and with IVH grade I or with IVH grade II-IV.

Gene symbol		Group without and IVH IN = 31(%)	Group with IVH grade II- IV N = 23(%)	P value	OR (95% CI)
eNOS *894G* > *T* (rs1799983)	**Genotype**				
	GG	19 (61,29)	8 (34,78)	—	References
	GT	9 (29,03)	13 (56,52)	0,040	3,431 (1,049–11,22)
	TT	3 (9,68)	2 (8,7)	1,000	1,583 (0,110–16,58)
	**Allele**				
	G	47 (75,81)	29 (63,04)	—	References
	T	15 (24,19)	17 (36,96)	0,222	1,837 (0,7333–4,605)
eNOS *−786T* > *C* (rs2070744)	**Genotype**				
	TT	13 (41,94)	6 (26,09)	—	References
	TC	15 (48,39)	14 (60,87)	0,398	2,022 (0,522–8,297)
	CC	3 (9,68)	3 (13,04)	0,726	2,167 (0,215–20,89)
	**Allele**				
	T	41 (66,13)	26 (56,52)	—	References
	C	21 (33,87)	20 (43,48)	0,414	1,502 (0,636–3,540)
EDN1 *5665G* > *T* (rs5370)	**Genotype**				
	GG	21 (67,74)	16 (69,57)	—	References
	GT	10 (32,26)	6 (26,09)	0,937	0,787 (0,193–3,029)
	TT	0 (0,00)	1 (4,35)	—	—
	**Allele**				
	G	52 (83,87)	38 (82,61)	—	References
	T	10 (16,13)	8 (17,39)	1,000	1,095 (0,340–3,413)

Results are expressed as absolute number of patients (percentage). The odds ratio (OR) and 95% confidence intervals (95% CI). GG denotes homozygosity for the G-encoded eNOS *894G* > *T* polymorphism variant; TT homozygosity for the T-encoded eNOS *894G* > *T* polymorphism variant; G*T* heterozygosity for eNOS *894G* > *T* polymorphism. TT denotes homozygosity for the T-encoded eNOS *−786T* > *C* polymorphism variant; CC homozygosity for the *C*-encoded eNOS *−786T* > *C* polymorphism variant; T*C* heterozygosity for eNOS *−786T* > *C* polymorphism. GG denotes homozygosity for the G-encoded EDN1 *5665G* > *T* polymorphism variant; TT homozygosity for the T-encoded EDN1 *5665G* > *T* polymorphism variant; GT heterozygosity for EDN1 *5665G* > *T* polymorphism. EDN1 -endothelin-1; eNOS- endothelial nitric oxide synthase.

**Table 4:** Genotype distribution of the polymorphisms in hypotensive and non-hypotensive infants with IVH grade II-IV.

Gene symbol		Group IVH grade II-IV without hypotension N = 15(%)	Group with IVH grade II- IV with hypotension N = 16(%)	P value	OR (95% CI)
eNOS *894G* > *T* (rs1799983)	**Genotype**				
	GG	7 (46,67)	6 (37,50)	—	References
	GT	7 (46,67)	8 (50,00)	1,000	1,333 (0,238–7,558)
	TT	1 (6,67)	2 (12,50)	1,000	2,333 (0,093–157,0)
	**Allele**				
	G	21 (70,00)	20 (62,50)	—	References
	T	9 (30,00)	12 (37,50)	0,724	1,4 (0,429–4,651)
eNOS *−786T* > *C* (rs2070744)	**Genotype**				
	TT	2 (13,33)	5 (31,25)	—	References
	TC	11 (73,33)	8 (50,00)	0,378	0,291 (0,023–2,475)
	CC	2 (13,33)	3 (18,75)	1,000	0,6 (0,029–13,19)
	**Allele**				
	T	15 (50,00)	18 (56,25)	—	References
	C	15 (50,00)	14 (43,75)	0,812	0,778 (0,255–2,369)
EDN1 *5665G* > *T* (rs5370)	**Genotype**				
	GG	11 (73,33)	10 (62,50)	—	References
	GT	4 (26,67)	5 (31,25)	1,000	1,375 (0,219–9,006)
	TT	0 (0,00)	1 (6,25)	—	—
	**Allele**				
	G	26 (86,67)	25 (83,33)	—	References
	T	4 (13,33)	7 (16,67)	0,587	1,82 (0,399–9,474)

Results are expressed as absolute number of patients (percentage). The odds ratio (OR) and 95% confidence intervals (95% CI). GG denotes homozygosity for the G-encoded eNOS *894G* > *T* polymorphism variant; TT homozygosity for the T-encoded eNOS *894G* > *T* polymorphism variant; G*T* heterozygosity for eNOS *894G* > *T* polymorphism. TT denotes homozygosity for the T-encoded eNOS *−786T* > *C* polymorphism variant; CC homozygosity for the *C*-encoded eNOS *−786T* > *C* polymorphism variant; TC heterozygosity for eNOS *−786T* > *C* polymorphism. GG denotes homozygosity for the G-encoded EDN1 *5665G* > *T* polymorphism variant; TT homozygosity for the T-encoded EDN1 *5665G* > *T* polymorphism variant; GT heterozygosity for EDN1 *5665G* > *T* polymorphism. EDN1 -endothelin-1; eNOS- endothelial nitric oxide synthase.

**Table 5:** Genotype distribution of the polymorphisms in infants treated due to acidosis with IVH grade II-IV.

Gene symbol		Group IVH grade II-IV without acidosis N = 14(%)	Group with IVH grade II- IV with acidosis N = 17(%)	P value	OR (95% CI)
eNOS *894G* > *T* (rs1799983)	**Genotype**				
	GG	5 (35,71)	8 (47,06)	—	References
	GT	8 (57,14)	7 (41,18)	0,685	0,547 (0,093–3,112)
	TT	1 (7,15)	2 (11,76)	1,000	1,250 (0,051–88,30)
	**Allele**				
	G	18 (64,29)	23 (67,65)	—	References
	T	10 (35,71)	11 (32,35)	0,991	0,861 (0,264–2,832)
eNOS *−786T* > *C* (rs2070744)	**Genotype**				
	TT	4 (28,57)	3 (17,65)	—	References
	TC	8 (57,14)	11 (64,71)	0,809	1,833 (0,229–15,89)
	CC	2 (14,29)	3 (17,65)	1,000	2,000 (0,121–37,87)
	**Allele**				
	T	16 (57,14)	17 (50,00)	—	References
	C	12 (42,86)	17 (50,00)	0,761	1,333 (0,435–4,113)
EDN1 *5665G* > *T* (rs5370)	**Genotype**				
	GG	11 (78,57)	10 (58,82)	—	References
	GT	3 (21,43)	6 (35,29)	0,579	2,200 (0,341–16,90)
	TT	0 (0,00)	1 (5,88)	—	—
	**Allele**				
	G	25 (89,29)	26 (76,47)	—	References
	T	3 (10,71)	8 (23,53)	0,328	2,564 (0,528–16,47)

Results are expressed as absolute number of patients (percentage). The odds ratio (OR) and 95% confidence intervals (95% CI). GG denotes homozygosity for the G-encoded eNOS *894G* > *T* polymorphism variant; TT homozygosity for the T-encoded eNOS *894G* > *T* polymorphism variant; G*T* heterozygosity for eNOS *894G* > *T* polymorphism. TT denotes homozygosity for the T-encoded eNOS −786T > *C* polymorphism variant; CC homozygosity for the *C*-encoded eNOS *−786T* > *C* polymorphism variant; TC heterozygosity for eNOS *−786T* > *C* polymorphism. GG denotes homozygosity for the G-encoded EDN1 *5665G* > *T* polymorphism variant; TT homozygosity for the T-encoded EDN1 *5665G* > *T* polymorphism variant; GT heterozygosity for EDN1 *5665G* > *T* polymorphism. EDN1 -endothelin-1; eNOS- endothelial nitric oxide synthase.

**Table 6:** Description of the polymorphisms in eNOS and EDN1genes.

Gene symbol	Polymorphism	Sequence of primers	Restriction enzyme	Products
eNOS	*894G* > *T (rs1799983*)	F 5′ AAggCAggAgACAgTggATgg A 3′ R 5′ CCC AgT CAA TCC CTT TggTgC TCA 3′	MboI	GG 248 bp GT 248, 158, 90 bp TT 158, 90 bp
eNOS	*−786T* > *C (rs2070744*)	F 5′ CCA CCC TgT CAT TCA gTg AC 3′ R 5 TCT CTgAgg TCT CgA AAT CA3′	PdiI	TT 296 bp TC 296, 220, 76 bp CC 220, 76 bp
EDN1	*5665G* > *T (rs5370*)	F 5′TCA TgA TCC CAA gCTgAAAgg CTA3′ R 5′ACC TTT CTT ggAATg TTT TgA AC3′	NheI	GG 203, 25 bp GT 228, 203, 25 bp TT 228 bp

GG denotes homozygosity for the G-encoded eNOS *894G* > *T* polymorphism variant; TT homozygosity for the T-encoded eNOS *894G* > *T* polymorphism variant; G*T* heterozygosity for eNOS *894G* > *T* polymorphism. TT denotes homozygosity for the T-encoded eNOS −786T > *C* polymorphism variant; CC homozygosity for the *C*-encoded eNOS *−786T* > *C* polymorphism variant; TC heterozygosity for eNOS *−786T* > *C* polymorphism. GG denotes homozygosity for the G-encoded EDN1 *5665G* > *T* polymorphism variant; TT homozygosity for the T-encoded EDN1 *5665G* > *T* polymorphism variant; G*T* heterozygosity for EDN1 *5665G* > *T* polymorphism. EDN1 - endothelin-1; eNOS-endothelial nitric oxide synthase.
